# Spatz: the time-of-flight neutron reflectometer with vertical sample geometry at the OPAL research reactor

**DOI:** 10.1107/S160057672201086X

**Published:** 2023-02-01

**Authors:** Anton P. Le Brun, Tzu-Yen Huang, Stewart Pullen, Andrew R. J. Nelson, James Spedding, Stephen A. Holt

**Affiliations:** a Australian Centre for Neutron Scattering, Australian Nuclear Science and Technology Organisation, New Illawarra Road, Lucas Heights, NSW 2234, Australia; b National Synchrotron Radiation Research Center, 101 Hsin-Ann Road, Hsinchu Science Park, Hsinchu, 30076, Taiwan; Montanuniversität Leoben, Austria

**Keywords:** neutron reflectometry, instrument commissioning, surfaces, interfaces, time of flight

## Abstract

The Spatz neutron beam instrument is the second time-of-flight neutron reflectometer to be installed at the OPAL research reactor. The instrument was formerly the V18 BioRef reflectometer at the BER-II reactor in Berlin and was transferred to Australia in 2016. Subsequently the instrument was re-installed in the neutron guide hall of the OPAL reactor at the end position of the CG2B cold-neutron guide and recommissioned. The instrument performance has not been compromised by the move, with reflectivity achieved down to 10^−7^ and good counting statistics within a reasonable time frame using a wavelength range of 2–20 Å. Several different samples at the solid–air interface and the solid–liquid interface have been measured to demonstrate the instrument’s capabilities.

## Abstract 2

The performance of the Spatz neutron reflectometer is demonstrated. It has a variable wavelength resolution to suit experimental needs for reflectometry at air–solid and solid–liquid interfaces.

## 1. Introduction

Neutron reflectometry is a technique to study the structure of surfaces and interfaces down to the nanoscale. In the specular regime (where angle of incidence = angle of reflection) information on the thickness and composition of different layers along the surface normal direction can be determined, and in some special cases the off-specular regime (angle of incidence ≠ angle of reflection) can provide information on in-plane structures (Torikai, 2011 ▸). Neutrons interact weakly with matter, making them highly penetrating and therefore an ideal radiation source for reflectometry, as this means buried interfaces or interfaces under liquid conditions can be measured and a range of different sample environments can be used (Lindner *et al.*, 2011 ▸).

The neutron scattering lengths of materials vary from isotope to isotope, as well as across elements, and the ability to discriminate between different isotopically labelled compounds is particularly advantageous. For example, in soft-matter and biosciences the difference in scattering length between hydrogen (*b*_H_ = −3.741 × 10^−5^ Å) and its isotope deuterium (*b*_D_ = 6.671 × 10^−5^ Å) is regularly exploited (Heinrich, 2016 ▸; Le Brun *et al.*, 2016 ▸).

Examples where neutron reflectivity has had a significant impact on scientific applications include colloid and surfactant chemistry (Penfold & Thomas, 2014 ▸), membrane structural biology (Lakey, 2019 ▸), polymer science (Braun *et al.*, 2017 ▸), electrochemistry and corrosion (Wood & Clarke, 2017 ▸), thin-film magnetism (Yusuf & Kumar, 2017 ▸), photovoltaic devices (Clulow *et al.*, 2014 ▸), pharmaceuticals (Lolicato *et al.*, 2019 ▸), and many more.

Neutron scattering is carried out at either reactor-based or spallation-based neutron facilities. Most neutron facilities will have a suite of instruments that can perform reflectometry experiments for the study of phenomena at surfaces and interfaces (Ambaye *et al.*, 2008 ▸; Ankner *et al.*, 2008 ▸; Campbell *et al.*, 2011 ▸; Cubitt & Fragneto, 2002 ▸; Devishvili *et al.*, 2013 ▸; Dura *et al.*, 2006 ▸; Khaydukov *et al.*, 2015 ▸; Webster *et al.*, 2011 ▸; Yamada *et al.*, 2011 ▸). At the neutron beam facility of the 20 MW OPAL (Open Pool Australian Light-water) research reactor (Sydney, Australia) there has been one operating reflectometer, known as Platypus (James *et al.*, 2011 ▸). The instrument has a horizontal sample geometry (vertical scattering geometry) that operates in time-of-flight mode and is a multi-purpose reflectometer for the study of samples at the gas–solid, solid–liquid or gas–liquid interfaces (free liquid surfaces). The instrument also has a capability for complete polarization analysis for studying magnetic systems (Saerbeck *et al.*, 2012 ▸). Platypus has been in user operation since 2008, and consistent oversubscription has meant there has been a need for a second reflectometer at the facility for several years.

This need was fulfilled by the transfer of an instrument from another facility to Australia. The V18 BioRef reflectometer was originally situated at the BER-II research reactor [Helmholtz Zentrum Berlin (HZB), Berlin, Germany] (Strobl *et al.*, 2011 ▸; Trapp *et al.*, 2016 ▸), and following the scheduled closure of the reactor the instrument was transferred to the ownership of the Australian Nuclear Science and Technology Organisation (ANSTO). The BioRef instrument was disassembled and shipped to Australia for re-assembly in the neutron guide hall of the OPAL research reactor (Le Brun & Pullen, 2021 ▸). On arrival at ANSTO the instrument was renamed Spatz, which is German for sparrow, continuing the ANSTO convention of naming neutron beam instruments after animals. This paper describes the results from the commissioning of the instrument to demonstrate its performance at its new location.

## 2. Description of the instrument

### 2.1. CG2B cold-neutron guide

Spatz is situated in the neutron guide hall of the OPAL research reactor and views the cold-neutron source which moderates neutrons using liquid deuterium at ∼23 K (Kennedy, 2006 ▸). The instrument occupies the end position of the CG2B cold-neutron guide which transports neutrons from the reactor face to the neutron guide hall. The guide from SwissNeutronics AG (Klingnau, Switzerland) was installed in two stages, with the first 23.35 m between the source and secondary shutter installed in 2013 (Klose *et al.*, 2016 ▸) and the remaining 31.15 m installed in 2018. The guide has dimensions of 50 mm (width) × 160 mm (height) with *m* = 3 coatings on the top and bottom and *m* = 2 and *m* = 2.5 on the left and right sides, respectively, and a radius of curvature of 1300 m. At 47.65 m from the source the guide then reduces to 30 mm (width) × 50 mm (height), which matches the characteristics needed for the Spatz instrument. The instrument views the top section of CG2B, allowing for a future instrument at this position that would view the bottom half of the guide. The coatings on this section are *m* = 3 on the top and bottom and *m* = 1 on the sides. At the end of the guide there is an oscillating attenuator which was designed and installed by ANSTO, and a beam monitor with an efficiency of 5 × 10^−6^ for neutrons with a wavelength of 4 Å (model 4560N, ORDELA Inc., Oak Ridge, Tennessee, USA). There is then a short section of guide followed by the instrument tertiary shutter.

### 2.2. Instrument hardware

**Figure 1:**
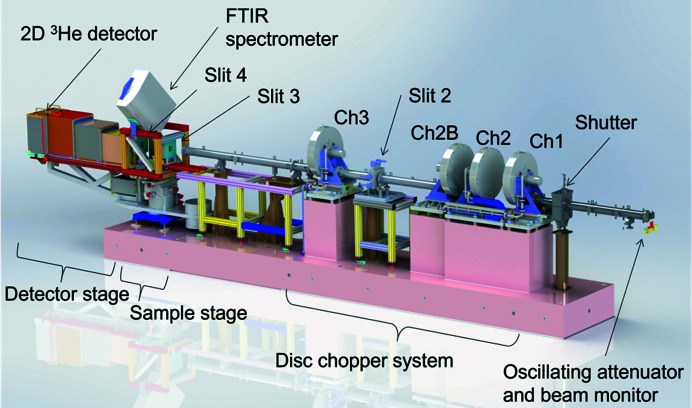
The layout of the Spatz neutron beam instrument, highlighting some of the major components. Shielding elements have been removed around the choppers, slits and guides for clarity.

All the major components of the instrument were retained during the transfer and installed in the neutron guide hall of the OPAL research reactor. The layout of the instrument can be seen in Fig. 1 ▸. Detailed descriptions of instrument hardware can be found in the reports by Strobl *et al.* (2011 ▸) and Trapp *et al.* (2016 ▸). The instrument operates under the time-of-flight (ToF) principle, similar to other ToF reflectometers at reactor sources (van Well & Fredrikze, 2005 ▸). Two disc choppers pulse the beam, and the time of flight from the choppers to the detector is measured to determine the neutron wavelength λ *via* the de Broglie relation,

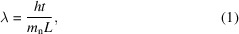

where *h* is the Planck constant, *m*_n_ is the mass of a neutron, *t* is the time of flight and *L* is the distance travelled, in this case the distance from the choppers to the detector. Therefore, the first major part after the instrument shutter is the rotating disc chopper system (Forschungszentrum Jülich GmbH, Jülich, Germany) that pulses the neutron beam. There are four boron carbide-coated disc choppers denoted 1, 2, 2B and 3, providing a fractional wavelength resolution (Δλ/λ) of 1–12%. Chopper 2 is on a translation stage that can vary the distance between choppers 1 and 2 from 80 to 480 mm, providing for a configurable wavelength resolution (Trapp *et al.*, 2016 ▸). The final chopper (chopper 3) is a frame-overlap chopper to remove long-wavelength neutrons and thus prevent frame overlap. The previous frame overlap mirror was discontinued and replaced with a straight section of guide while the instrument was at the BER-II reactor, and frame overlap suppression is only from chopper 3 (Trapp *et al.*, 2016 ▸). There are three sets of four-bladed slit systems (JJ X-ray A/S, Hoersholm, Denmark), located between choppers 2B and 3, and pre- and post-sample, which are denoted 2, 3 and 4, respectively (as slit 1 had been discontinued while the instrument was at the BER-II reactor) (Fig. 1 ▸). Slits 2 and 3 define the angular resolution (ΔΩ/Ω) and illuminated footprint, whilst the final slit (slit 4) behind the sample prevents the transmitted beam from reaching the detector. The guides, chopper system and slit 2 are all enclosed within suitable lead and borated shielding to keep the combined γ-ray and neutron equivalent dose rate below 3 µSv h^−1^ at the enclosure perimeter when the shutters are open.

The sample stage consists of stacked motion stages to rotate the sample (angle of incidence Ω) and detector angle (2θ), translate the sample across the beam (with a coarse translation stage and fine translation stage), and tilt the sample (ψ angle). Samples are mounted using a vertical sample geometry, giving the instrument a horizontal scattering geometry. An additional height stage has been added that can hold multiple samples as well as provide height adjustment for aligning samples. Finally, the detector is a DENEX-TN300 ^3^He delay line two-dimensional detector (Denex GmbH, Lueneburg, Germany) which can also provide for an off-specular reflectometry capability.

### 2.3. Instrument software

Instrument software controls have been harmonized with all the other instruments at the facility through a client server architecture known as the *SICS* instrument control software (Heer *et al.*, 1997 ▸). *SICS* controls all motions and sample environments and starts data acquisitions. A separate ‘histogram memory server’ (*DAS*) performs the data acquisition. The *DAS* generates histograms for all the time and spatial information for each neutron event detected, and an event file is also recorded for each measurement to permit real-time measurements. *SICS* saves the collected data in a NeXus file (Könnecke *et al.*, 2015 ▸) along with all the instrument settings used to collect the data. This NeXus file can then be processed further by the user to obtain the reflectivity profiles needed for data analysis. Sitting above *SICS* is the *GumTree* graphical user interface (Lam *et al.*, 2006*a* ▸,*b* ▸), which is written in the Java programming language using the Eclipse Rich Client Platform. Scientists use *GumTree* to control the instrument in an integrated and user-friendly way to carry out experiments on Spatz.

Data reduction is done in a Jupyter notebook using the *refnx* package (Nelson & Prescott, 2019 ▸) (see Section S2 in the supporting information for the format of the notebook). The data reduction accounts for possible event-mode reduction for real-time measurements, accounts for detector efficiency and converts time of flight to wavelength, which is then used to calculate the momentum transfer *Q* according to

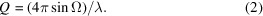

Here Ω is the angle of incidence. The data reduction also re-bins in wavelength, subtracts the background, integrates the specular signal to create a wavelength spectrum, divides the reflected and direct beams to produce a reflectivity curve, stitches the two or three angles together at appropriate overlap regions, and scales the data so that any critical edge is unity. The reduced data are output in four columns of *Q* (momentum transfer in units of Å^−1^), *R* (reflectivity), d*R* (standard deviation) and d*Q* [instrument resolution, which is accounted for according to Nelson (2013 ▸)].

### 2.4. Sample environments and preparation

A range of sample environments are available for use on the instrument. These include the following:

(i) Low background/low volume (280 µl) solid–liquid cells that fit 10 cm diameter round silicon wafers which have silicon backing plates, inlet and outlet tubes for solvent exchange, and aluminium temperature jackets for temperature control through a circulating water bath.

(ii) A high-performance liquid chromatography (HPLC) pump (Azura P6.1L, KNAUER Wissenschaftliche Geräte GmbH, Berlin, Germany) for automatic solvent exchange, such as changing D_2_O/H_2_O isotopic contrasts.

(iii) A sample changer that can hold multiple samples for automated sample changing by moving samples vertically in or out of the beam. The sample changer can hold three of the solid–liquid cells mentioned above and several samples at the air–solid interface under ambient conditions.

(iv) An atmospheric chamber with temperature control which can be used for different gas environments or vacuum. This chamber can also be connected to either the static or dynamic vapour delivery systems (Clulow *et al.*, 2017 ▸).

(v) Electrochemical cells for applying electrical potentials, cyclic voltammetry and impedance spectroscopy.

The original attenuated total reflection Fourier transform infrared (IR) spectrometer setup for simultaneous IR spectroscopy and neutron reflectometry is still available (Strobl *et al.*, 2011 ▸). A fuller range of sample environments can be found in the facility sample environment manual (ACNS Sample Environment Group, 2021 ▸). There is also a full range of equipment for sample preparation and/or pre-characterization or optimization, including a spin coater, an ozone cleaner, a preparative Langmuir trough for Langmuir–Blodgett/Langmuir–Schaefer dipping, a quartz crystal microbalance with dissipation (QCM-D), an X-ray reflectometer and an imaging variable angle spectroscopic ellipsometer, as well as standard laboratory equipment.

## 3. Beam characteristics

**Figure 2:**
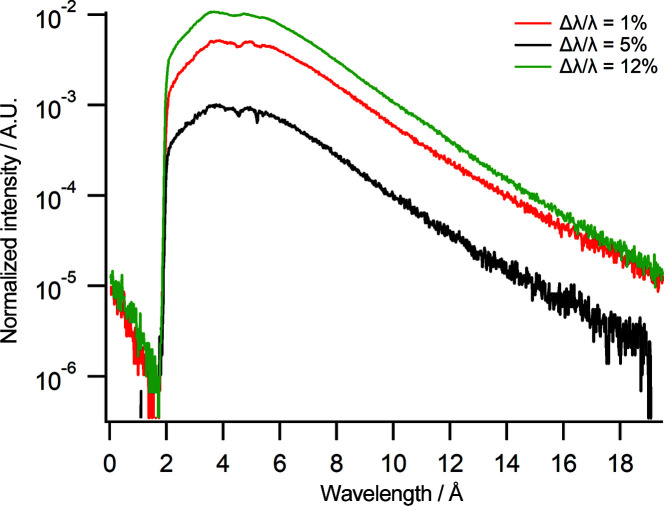
Neutron spectra at different chopper settings. Black: Δλ/λ = 1% (chopper pairing 1 and 2 with chopper distance set to 80 mm). Red: Δλ/λ = 5% (chopper pairing 1 and 2 with chopper distance set to 480 mm). Green: Δλ/λ = 12% (chopper pairing 1 and 2B with chopper distance fixed at 960 mm). The intensity is normalized to the beam monitor counts.

The flux at the end of the CG2B cold-neutron guide as measured by gold foil activation is 1.01 × 10^10^ n cm^−2^ s^−1^ averaged over a grid of 3 × 3 gold foils which were equally separated over the area of the guide ending at 19.1 MW reactor power. The spectra displayed in Fig. 2 ▸ were measured on Spatz at three different chopper settings, corresponding to wavelength resolutions of Δλ/λ = 1, 5 and 12%, and are normalized to the beam monitor counts at the front of the instrument. For all the different chopper pairing/distance settings the choppers were operating at a speed of 25 Hz with a slit setting of slits 2 and 3 = 0.2 mm and counting times of 3600 s. Each of the spectra in Fig. 2 ▸ has essentially the same shape with a spectrum of 1.8–19.6 Å, making for a usable wavelength range from approximately 2.8 to 18 Å. As expected, the intensity increases with resolution, with Δλ/λ = 12% having the highest counts. There is a variation in the spectrum with Δλ/λ = 12% at longer wavelengths as the resolution is no longer constant at these wavelengths with the chopper pairing of 1 and 2B. The spectrum has two deviations in intensity at 4.6 and 5.2 Å which are due to aluminium windows along the beamline.

The wavelength is calibrated using the principles described by Campbell *et al.* (2011 ▸) using a Bragg mirror consisting of 25 bilayers of nickel and titanium (see Section 4.1 for further details of the Bragg mirror). First, the sample-to-detector distance is calibrated by measuring the difference in pixel position between the reflected and direct beams at different sample-to-detector distances. This difference is plotted as a function of detector distance and the intercept is the true sample-to-detector distance = 0 m. The reflection from the Bragg mirror is then measured at an angle of incidence = 3.8° at different sample-to-detector distances, and the time bin positions of each peak are converted to seconds and plotted as a function of detector distance. The wavelength that corresponds to each peak can then be determined by the product of the gradient and *h*/*m*_n_ (3955.8). The next step in calibrating the wavelength involves determining the phase offsets for the chopper pairing. Using the peak that corresponds to a wavelength of ∼6–7 Å, the phase on the secondary chopper that is required to extinguish that peak is calculated as

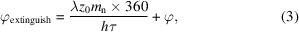

where *z*_0_ is the distance between the chopper pairing, τ is the chopper period in seconds and φ is the nominal phase difference between the discs. The phase to extinguish the peak at ∼6–7 Å is then found experimentally by measuring the intensity of the peak at different secondary chopper phases using a fixed detector distance until the peak is extinguished. The intensity decreases linearly with increasing secondary chopper phase, and the intercept is the actual phase required to extinguish the peak. The difference between the calculated and experimental values is the phase offset needed for the secondary chopper to ensure that the choppers are optically blind. Finally, the primary chopper phase offset is determined. This is done by measuring the spectrum of the Bragg mirror at 3.8° and then varying the phase offset of the primary chopper until the sum of the squared differences between the actual peak wavelengths and where they should be is minimized.

**Figure 3:**
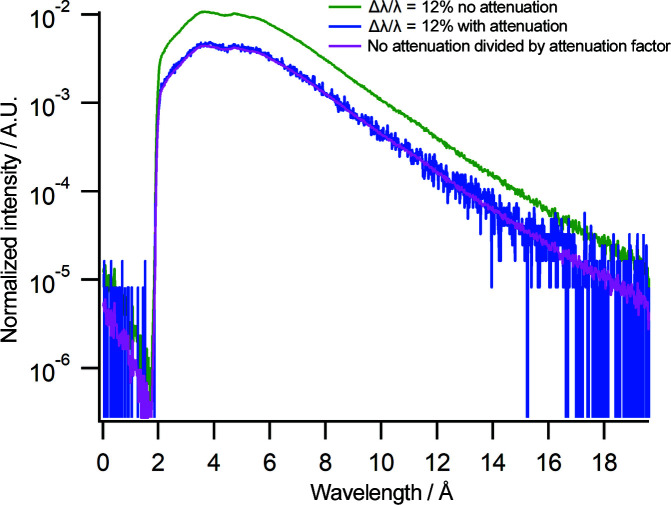
Neutron wavelength spectra at Δλ/λ = 12% (chopper pairing 1 and 2B) without (green) and with (blue) attenuation. The magenta line shows the spectrum without attenuation divided by the attenuation factor. The intensity is normalized to the beam monitor counts.

An oscillating attenuator has been installed between the end of the CG2B guide and the beam monitor (Fig. 1 ▸) to reduce the flux in a wavelength-independent manner. The use of the oscillating attenuator will prevent detector damage and counting loss when taking direct-beam measurements for large slit settings. The attenuator is a boron carbide fixed slit that oscillates across the beam, reducing the neutron flux at the detector and the beam monitor. Fig. 3 ▸ shows the spectrum at Δλ/λ = 12% using the same slit settings and counting times as described for Fig. 2 ▸ with and without the oscillating attenuator activated. As can be seen in the blue spectrum, the attenuator reduces the neutron flux. The attenuation factor is 55. The magenta line is the spectrum without attenuation but reduced by the attenuation factor. This spectrum overlays the spectrum with the oscillating attenuator activated, demonstrating that the attenuation is wavelength independent.

## 4. Sample reflectivity data

### 4.1. Bragg mirror

**Figure 4:**
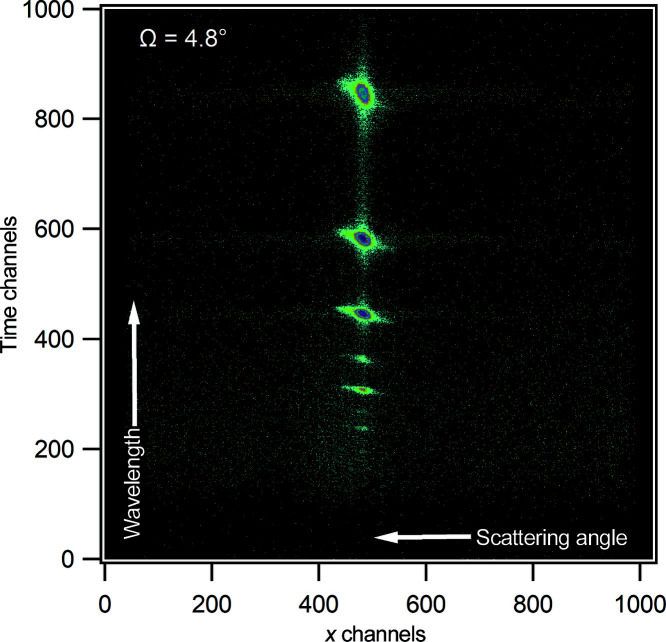
A raw two-dimensional detector image of reflection from a Bragg mirror at an angle of incidence of 4.8°. The image shows the specular (in the vertical direction) and off-specular reflection. The time channels are related to wavelength and the *x* channels to scattering angle.

**Figure 5:**
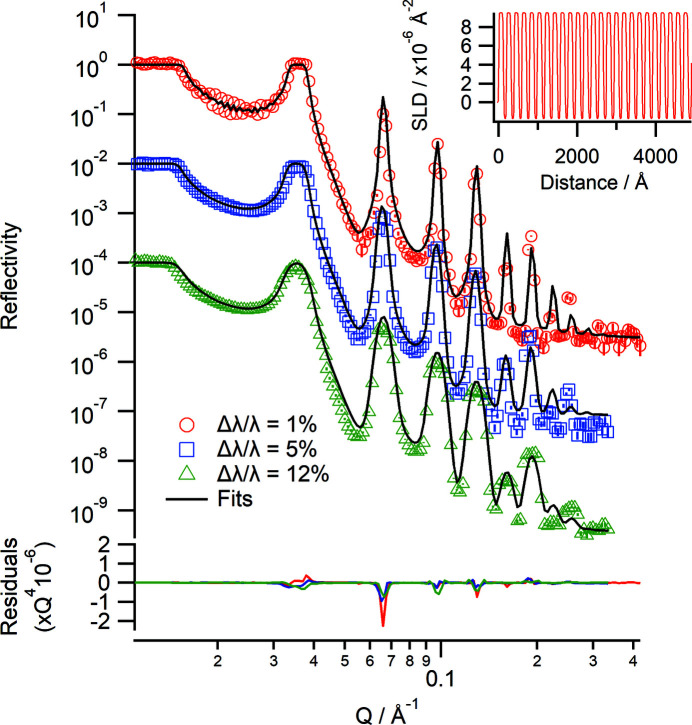
Neutron reflectivity profiles of a Bragg mirror consisting of 25 bilayers of Ni/Ti at Δλ/λ = 1% (red), Δλ/λ = 5% (blue) and Δλ/λ = 12% (green). The solid lines are the fits to each data set. The data are offset for clarity. The lower panel shows the residuals between the data and fits normalized to *Q* ^4^. (Inset) The corresponding real-space SLD profile from the fit in the Δλ/λ = 1% case.

Several different samples have been measured to test the performance of the instrument. The first measurement is a Bragg mirror which consists of 25 bilayers of nickel and titanium deposited on float glass. The reflectivity gives rise to several Bragg peaks corresponding to the spacing of the bilayers. Fig. 4 ▸ shows a two-dimensional detector image of the data at an angle of incidence of 4.8° (using a chopper setting of Δλ/λ = 5%), where the Bragg peaks can be clearly seen along the specular ridge. The off-specular scattering around the peaks can also be observed. The Bragg mirror was measured at angles of incidence of 1.0 and 4.8° at different chopper configurations using an illuminated footprint of 30 mm (Fig. 5 ▸). For the Δλ/λ = 1% chopper setting, the Kiessig fringes associated with the total thickness of all 25 bilayers are observable and nine Bragg peaks can be observed at all chopper settings used. The data were fitted individually in *refnx* using a multilayer model (Nelson & Prescott, 2019 ▸). The thickness of the nickel layers is 117 (±1) Å and that of the titanium layers is 79 (±1) Å across all three data sets, in good agreement with previously published values (James *et al.*, 2011 ▸). The fitted values were also confirmed by simultaneously fitting the three data sets using the same model as the individual fits.

### 4.2. Air–solid interface

Several different substrates were measured at the air–solid interface, namely silicon, quartz and sapphire. The scattering length density (SLD) and critical edge (*Q*_c_) for each substrate are given in Table 1 ▸. The critical edge is the point below which total external reflection occurs and above which reflectivity will decrease with a *Q* ^−4^ dependence. The critical edge for a given substrate/subphase combination is calculated as

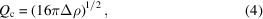

where Δρ is the difference in SLD between the substrate and subphase.

**Figure 6:**
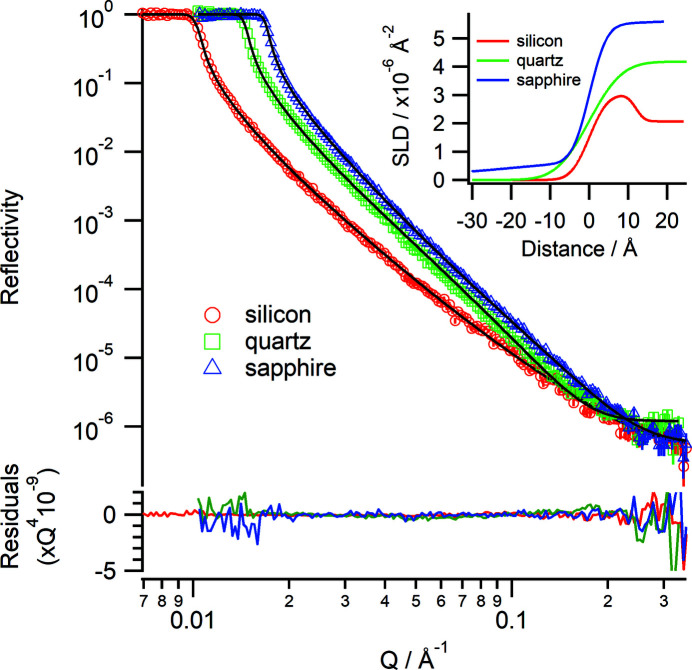
Neutron reflectivity profiles (Δλ/λ = 5%) from a silicon wafer (red), quartz wafer (green) and sapphire wafer (blue) in air with fits (solid lines). The lower panel shows the residuals between the data and fits normalized to *Q* ^4^. (Inset) The corresponding real-space SLD profiles.

Fig. 6 ▸ shows the reflectivity for each substrate and the corresponding fits. Each substrate was measured using a 30 mm footprint and an angular resolution ΔΩ/Ω = 3%, with three angles of incidence of 0.6, 1.7 and 4.0° for silicon and two angles of incidence of 0.9 and 4.0° for quartz and sapphire. The chopper settings were Δλ/λ = 5% at 25 Hz. For the silicon wafer a thin native oxide layer was used during modelling; for the sapphire substrate a thin low-density hydrocarbon layer was needed to obtain a good fit (it could not be removed even with cleaning). The *Q*_c_ values and the fitted SLD (Fig. 6 ▸ inset) are in good agreement with the values in Table 1 ▸, demonstrating that the wavelength calibrations are good. A good background is achieved, with reflectivity down to 10^−7^ for a total data collection time of 1.5 h.

**Figure 7:**
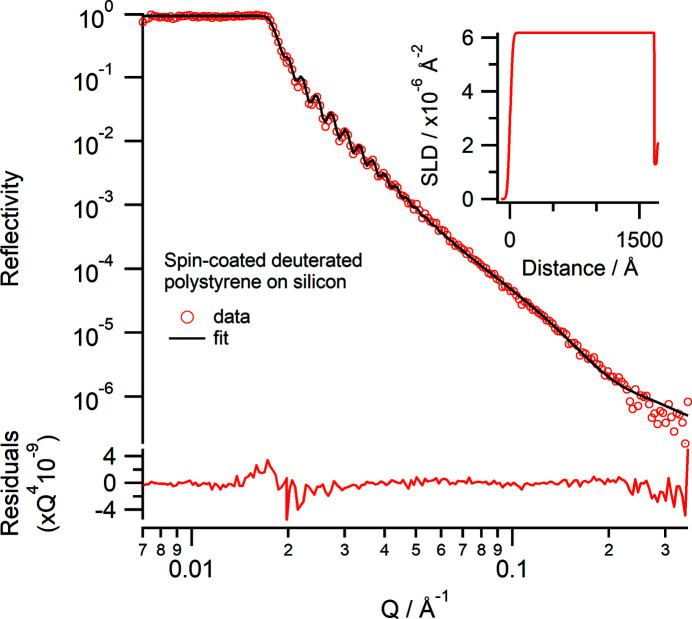
Neutron reflectivity (Δλ/λ = 5%) profile of a spin-coated deuterated polystyrene layer on silicon in air. The lower panel shows the residuals between the data and fit normalized to *Q* ^4^. (Inset) The corresponding real-space SLD profile to the fit.

A silicon wafer was spin-coated with deuterated polystyrene (d_8_-polystyrene) from toluene. The spin-coating conditions were 500 r min^−1^ for 10 s and then 2000 r min^−1^ for 60 s using 20 mg ml^−1^ d_8_-polystyrene (*M*_n_ 215 000, *M*_w_ 236 500, *M*_w_/*M*_n_ = 1.10) in toluene. The film was measured using the same instrument conditions as for the silicon wafer in Section 4.2. The d_8_-polystyrene layer showed good Kiessig fringes (Fig. 7 ▸). The data were fitted using a slab model with a fitted SLD of 6.18 (±0.01) × 10^−6^ Å^−2^, a thickness of 1672 (±1) Å and a roughness of 24.4 (±0.3) Å for the d_8_-polystyrene (Fig. 7 ▸ inset). A thin layer of thickness 29 (±1) Å with a low SLD of 1.29 (±0.02) × 10^−6^ Å^−2^ and a roughness of 1.1 (±0.5) Å between the silicon substrate and d_8_-polystyrene needed to be included for the best fit; this layer could be a region enriched with residual toluene, because the sample was not thermally annealed, as has been previously observed (Perlich *et al.*, 2009 ▸).

### 4.3. Solid–liquid interface

**Figure 8:**
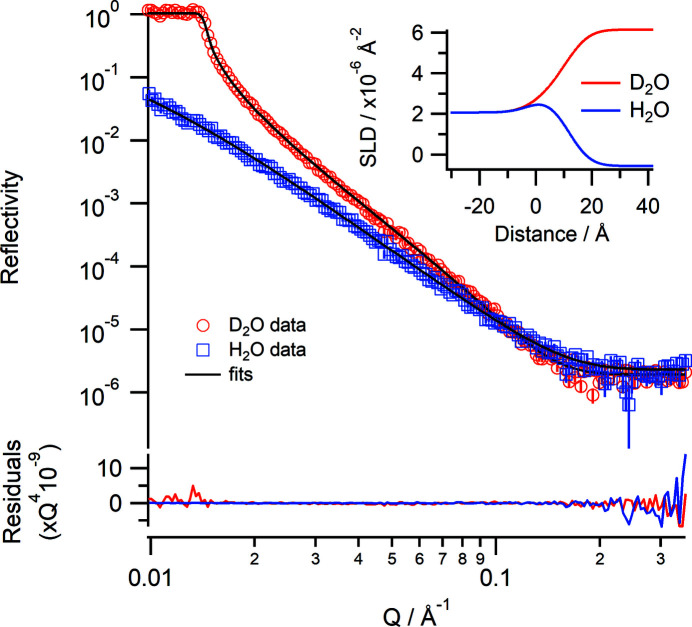
Neutron reflectivity profiles of a silicon wafer in D_2_O (red) and H_2_O (blue) and the simultaneous fits (black lines). The lower panel shows the residuals between the data and fits normalized to *Q* ^4^. (Inset) The corresponding real-space SLD profiles.

Measurements on Spatz can also be performed at the solid–liquid interface. Fig. 8 ▸ shows the reflectivity of a silicon wafer using a D_2_O and an H_2_O subphase. Measurements were taken using angles of incidence of 0.85 and 4.0° using a 30 mm footprint and ΔΩ/Ω = 3% and Δλ/λ = 5% with a total counting time of 75 min for each contrast. The data show that the silicon–D_2_O critical edge is *Q*_c_ = 0.0141 Å^−1^ and the fitted SLD of the bulk D_2_O is 6.14 (±0.02) × 10^−6^ Å^−2^, which are close to the theoretical values of 0.0147 Å^−1^ and 6.36 × 10^−6^ Å^−2^, respectively. The variation is probably down to isotopic impurities in the D_2_O, the source of which is likely to be incomplete mixing in the cell. To demonstrate the performance of Spatz using samples at the solid–liquid interface two different samples were measured: a thick film of polystyrene on silicon and a thin film composed of a phospho­lipid bilayer.

**Figure 9:**
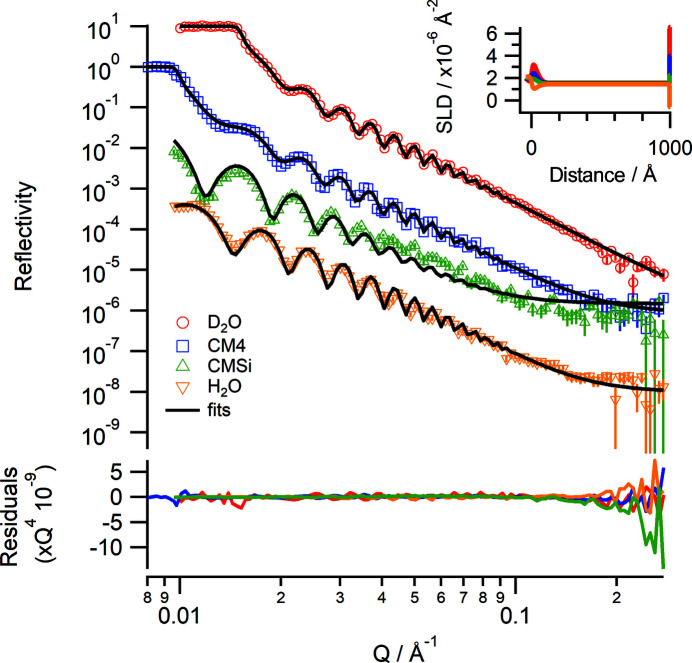
Neutron reflectivity profiles of a hydrogenous polystyrene layer (thickness = 971 Å) spin-coated onto silicon at the solid–liquid interface under four different isotopic contrasts of D_2_O (red), CM4 (blue), CMSi (green) and H_2_O (yellow). The data points are the collected data and the solid lines are a co-refined fit using a slab model. Data are offset for clarity. The lower panel shows the residuals between the data and fits normalized to *Q* ^4^. (Inset) The corresponding real-space SLD profiles.

A hydrogenous polystyrene film (*M*_n_ 170 000, *M*_w_ 350 000, *M*_w_/*M*_n_ = 2.06) was spin-coated from a solution at 20 mg ml^−1^ in toluene onto a 100 mm diameter silicon wafer at 500 r min^−1^ for 10 s and then 4000 r min^−1^ for 60 s, and measured in liquid under four different isotopic contrasts: D_2_O (SLD = 6.36 × 10^−6^ Å^−2^), CM4 (contrast matched to 4) (SLD = 4.00 × 10^−6^ Å^−2^), CMSi (contrast matched to silicon) (SLD = 2.07 × 10^−6^ Å^−2^) and H_2_O (SLD = −0.56 × 10^−6^ Å^−2^). The choice of using a hydrogenous polystyrene in this case was to achieve a good contrast between the polymer film and the D_2_O. Reflectivity data were measured using angles of incidence of 0.85 and 3.5° with a 40 mm footprint at ΔΩ/Ω = 3% and Δλ/λ = 5%. An additional angle of incidence at 0.5° was measured for the CM4 contrast to ensure that data around the critical edge (*Q*_c_ = 0.00985 Å^−1^) were collected. The solvent contrast changes were automated using the HPLC pump so that all contrasts were collected within approximately 8 h. Fig. 9 ▸ shows the collected reflectivity data and corresponding fits that were co-refined. Well resolved Kiessig fringes are observed, and the thickness of the polystyrene layer was determined to be 971 (±2) Å using a slab model with a fitted SLD of 1.48 (±0.01) × 10^−6^ Å^−2^, solvent volume fraction of 0.015 (±0.003) and roughness of 38 (±2) Å. There is also a thin solvated layer between the silicon and polystyrene of thickness 24 (±2) Å and roughness 9 (±1) Å.

**Figure 10:**
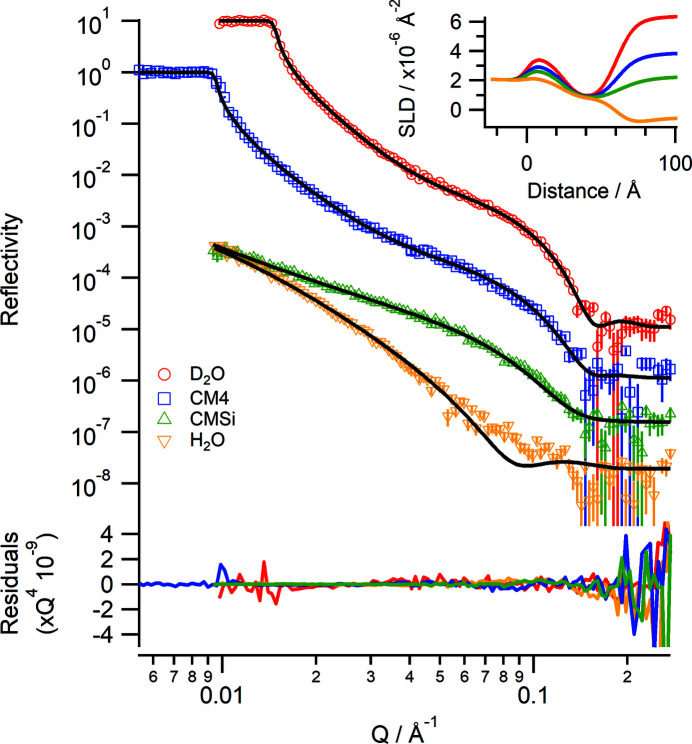
Neutron reflectivity profiles of a DMPC bilayer at 310 K deposited onto a silicon wafer by vesicle rupture under different isotopic solvent contrasts. Red D_2_O, blue CM4, green CMSi, yellow H_2_O and black lines: fitted data. Data are offset for clarity. The lower panel shows the residuals between the data and fits normalized to *Q* ^4^. (Inset) The corresponding real-space SLD profile.

A phospholipid bilayer consisting of 1,2-dimyristoyl-*sn*-glycero-3-phosphocholine (DMPC) was deposited onto silicon using the vesicle rupture method (Johnson *et al.*, 1991 ▸) and measured under four isotopic contrasts of D_2_O, CM4, CMSi and H_2_O, each phosphate buffered with saline (137 m*M* NaCl, 3 m*M* KCl, 10 m*M* sodium phosphate pH 7.6). The instrument conditions were the same as for the polystyrene sample in Fig. 9 ▸, and the changes in contrast were also controlled under the HPLC pump. The sample cell was connected to a circulating water bath set to a temperature of 319 K, which is above the phase transition temperature of DMPC (*T*_m_ = 310 K). Fig. 10 ▸ shows the collected reflectivity data and corresponding fits. The single broad fringe in the D_2_O contrast indicates that a well formed bilayer has been deposited. The data were co-refined using the *Lipid Leaflet* component within *refnx* (Holt *et al.*, 2022 ▸) using volumes of 782 and 319 Å^3^ for the tails and headgroups, respectively. The fitted values for the bilayer are a total thickness of 35.6 (±1.2) Å (headgroup thickness = 5.2 Å and tail thickness = 12.6 Å for each leaflet), a lipid volume fraction of 0.809 and an area per lipid of 62.2 (±0.6) Å^2^. These values correspond well to published values from X-ray diffraction data for a stacked DMPC bilayer system in the fluid (*L*α) phase at 303 K of 36.0 Å for bilayer thickness and 59.6 Å^2^ for area per lipid (Nagle & Tristram-Nagle, 2000 ▸).

## 5. Summary

The V18 BioRef instrument was successfully transferred from the BER-II reactor to become the Spatz instrument at the OPAL reactor, keeping excellent instrument performance. Several different test samples have been measured, demonstrating the instrument’s performance and capabilities for measuring reflectivity samples under different experimental conditions. The motion, software and safety controls have been integrated into ANSTO systems whilst still retaining the original hardware elements of the instrument.

Spatz complements the existing reflectometer known as Platypus, which has a horizontal sample geometry making it suitable for measuring free-liquid surfaces. Spatz, with its vertical sample geometry, can undertake experiments at the air/gas–solid interface and at the solid–liquid interface using sample environments that are not available to Platypus. Spatz also has a larger detector rotation, allowing for wide-angle diffraction on suitable samples such as stacked lipid bilayers.

Spatz is a valuable addition to the neutron scattering facilities at the OPAL research reactor, increasing the capacity for neutron reflectometry experiments for soft-matter and biomolecular sciences. The instrument is currently available to receive proposals via the neutron scattering user portal at https://portal.ansto.gov.au/.

## Supplementary Material

Details of data files, data reduction software and fitting models. DOI: 10.1107/S160057672201086X/xx5008sup1.pdf

Raw data files, code for data reduction and model files for data fitting: https://doi.org/10.5281/zenodo.6829895

## Figures and Tables

**Table 1:** Scattering length densities and *Q*_c_ values against air for the substrates used in Fig. 6 ▸

Substrate	SLD (×10^−6^ Å^−2^)	*Q*_c_ (Å^−1^)
Silicon	2.07	0.0102
Quartz	4.23	0.0146
Sapphire	5.67	0.0169
