# Supplementary material for: Spatz: the time-of-flight neutron reflectometer with vertical sample geometry at the OPAL research reactor
Source: J Appl Crystallogr. 2023 Feb 1;56(Pt 1):18–25. doi: 10.1107/S160057672201086X (PMC9901927; doi:10.1107/S160057672201086X)
Supplement: Supplementary file 1 [file j-56-00018-sup1.pdf]

JOURNAL OF
APPLIED
CRYSTALLOGRAPHY

Volume 56 (2023)

Supporting information for article:

Spatz: the time-of-flight neutron reflectometer with vertical sample geometry at the OPAL research reactor

Anton Le Brun, Tzu-Yen Huang, Stewart Pullen, Andrew Nelson, James Spedding and Stephen Holt

The raw data files, code for data reduction, and model files for data fitting can be found at https://doi.org/10.5281/zenodo.6829895. This supporting information provides information on what the files are and how to use them.

S1. Data file descriptions

The raw data files are included with the manuscript. Supplementary Table S1 below provides a description for each NeXus data file (SPZ000XXXX.nx.hdf) which can be reduced to produce the final reflectivity as shown in the manuscript. Instructions for data reduction are described in section S2 of the supporting information.

Table S1 Description of each NeXus data file used in this work.

File number	Description	Angle of incidence
SPZ0000745	Bragg mirror at $\Delta\lambda/\lambda \sim 1\%$	0.9°
SPZ0000746	Bragg mirror at $\Delta\lambda/\lambda \sim 1\%$	4.8°
SPZ0000747	Direct beam measurement for Bragg mirror at $\Delta\lambda/\lambda \sim 1\%$ at 0.9° settings	n/a
SPZ0000748	Direct beam measurement for Bragg mirror at $\Delta\lambda/\lambda \sim 1\%$ at 4.8° settings	n/a
SPZ0000610	Bragg mirror at $\Delta\lambda/\lambda \sim 5\%$	1.0°
SPZ0000611	Bragg mirror at $\Delta\lambda/\lambda \sim 5\%$	4.0°
SPZ0000612	Direct beam measurement for Bragg mirror at $\Delta\lambda/\lambda \sim 5\%$ at 1.0° settings	n/a
SPZ0000613	Direct beam measurement for Bragg mirror at $\Delta\lambda/\lambda \sim 5\%$ at 4.0° settings	n/a
SPZ0000773	Bragg mirror at $\Delta\lambda/\lambda \sim 12\%$	0.9°
SPZ0000774	Bragg mirror at $\Delta\lambda/\lambda \sim 12\%$	3.8°
SPZ0000775	Direct beam measurement for Bragg mirror at $\Delta\lambda/\lambda \sim 12\%$ at 0.9° settings	n/a

SPZ0000776	Direct beam measurement for Bragg mirror at $\Delta\lambda/\lambda \sim 12\%$ at 3.8° settings	n/a
SPZ0000526	Silicon wafer	0.6°
SPZ0000527	Silicon wafer	1.7°
SPZ0000528	Silicon wafer	4.0°
SPZ0000529	Direct beam measurement for silicon wafer at 0.6° settings	n/a
SPZ0000530	Direct beam measurement for silicon wafer at 1.7° settings	n/a
SPZ0000531	Direct beam measurement for silicon wafer at 4.0° settings	n/a
SPZ0000581	Quartz wafer	0.9°
SPZ0000582	Quartz wafer	4.0°
SPZ0000591	Sapphire wafer	0.9°
SPZ0000592	Sapphire wafer	4.0°
SPZ0000593	Direct beam measurement for quartz and sapphire wafers at 0.9° settings	n/a
SPZ0000594	Direct beam measurement for quartz and sapphire wafers at 4.0° settings	n/a
SPZ0000675	D_8-polystyrene in air on silicon	0.6°
SPZ0000676	D_8-polystyrene in air on silicon	1.7°
SPZ0000677	D_8-polystyrene in air on silicon	4.0°
SPZ0000678	Direct beam measurement for D_8-polystyrene in air on silicon at 0.6° settings	n/a
SPZ0000679	Direct beam measurement for D_8-polystyrene in air on silicon at 1.7° settings	n/a
SPZ0000680	Direct beam measurement for D_8-polystyrene in air on silicon at 4.0° settings	n/a
SPZ0000665	Silicon-D_2O	0.85°
SPZ0000666	Silicon-D_2O	4.0°
SPZ0000667	Silicon-H_2O	0.85°
SPZ0000668	Silicon-H_2O	4.0°
SPZ0000658	Direct beam measurements for silicon-D_2O/H_2O at 0.85° settings	n/a
SPZ0000659	Direct beam measurements for silicon-D_2O/H_2O at 4.0° settings	n/a
SPZ0003493	Spin coated h-polystyrene on silicon in D_2O	0.85°

SPZ0003494	Spin coated h-polystyrene on silicon in D_2O	3.5°
SPZ0003495	Spin coated h-polystyrene on silicon in CM4	0.5°
SPZ0003496	Spin coated h-polystyrene on silicon in CM4	0.85°
SPZ0003497	Spin coated h-polystyrene on silicon in CM4	3.5°
SPZ0003498	Spin coated h-polystyrene on silicon in CMSi	0.85°
SPZ0003499	Spin coated h-polystyrene on silicon in CMSi	3.5°
SPZ0003500	Spin coated h-polystyrene on silicon in H_2O	0.85°
SPZ0003501	Spin coated h-polystyrene on silicon in H_2O	3.5°
SPZ0003473	DMPC bilayer on silicon at 37°C in D_2O	0.85°
SPZ0003474	DMPC bilayer on silicon at 37°C in D_2O	3.5°
SPZ0003475	DMPC bilayer on silicon at 37°C in CM4	0.5°
SPZ0003476	DMPC bilayer on silicon at 37°C in CM4	0.85°
SPZ0003477	DMPC bilayer on silicon at 37°C in CM4	3.5°
SPZ0003478	DMPC bilayer on silicon at 37°C in CMSi	0.85°
SPZ0003479	DMPC bilayer on silicon at 37°C in CMSi	3.5°
SPZ0003480	DMPC bilayer on silicon at 37°C in H_2O	0.85°
SPZ0003481	DMPC bilayer on silicon at 37°C in H_2O	3.5°
SPZ0003470	Direct beam for h-polystyrene and DMPC bilayer at 0.5° settings	n/a
SPZ0003471	Direct beam for h-polystyrene and DMPC bilayer at 0.85° settings	n/a
SPZ0003472	Direct beam for h-polystyrene and DMPC bilayer at 3.5° settings	n/a

S2. Description of the reduction code

The reduction code is within *refnx* in a Jupyter notebook format within a Conda environment and is called Spatz_reduction_demo.ipynb. Instructions and how to set-up the required conda environments and which features are needed can be found at: https://refnx.readthedocs.io/en/latest/installation.html. Below is a description of the steps needed to reduce the data once the needed computing environments have been set-up.

The first steps are to import the required modules, define the directory where the data files are, and import the manual beam finder.

```
%matplotlib notebook
%gui qt
import numpy as np
import matplotlib.pyplot as plt
import os.path


from refnx.reduce import SpatzNexus, SpatzReduce, catalogue, reduce_stitch
from refnx.reduce.manual_beam_finder import ManualBeamFinder
import refnx.util.ErrorProp as EP
from refnx.util import q
```

```
# data_folder = os.path.join('L:', 'data', 'cycle', 'current', 'sics')
# print(data_folder)

data_folder = './'
```

```
mbf = ManualBeamFinder()
```

There is also an option to catalogue the data and list the various settings.

```
catalogue(990, 1003, data_folder=data_folder, prefix='SPZ')
```

Next define which data files are the direct beams (db0, db1, etc.) and the reflected beam data (red0, red1, etc.) files. The final line defines the scale factor to use. Using the manual beam finder will create a pop-up window where the specular peak (foreground) and background regions can be manually defined.

```
db0 = SpatzReduce('SPZ0000977.nx.hdf', data_folder=data_folder)
db1 = SpatzReduce('SPZ0000978.nx.hdf', data_folder=data_folder)
#db2 = SpatzReduce('SPZ0000978.nx.hdf', data_folder=data_folder)
```

```
red0, reduction0 = db0.reduce('SPZ0001039.nx.hdf', rebin_percent=3.4, manual_beam_find=mbf, peak_pos=-1)
red1, reduction1 = db1.reduce('SPZ0001041.nx.hdf', rebin_percent=3.4, manual_beam_find=mbf, peak_pos=-1)
#red2, reduction2 = db2.reduce('SPZ0001041.nx.hdf', rebin_percent=3.0, manual_beam_find=mbf, peak_pos=-1)
red0[0].scale(1.05)
```

The data can also be plotted at this point, which is useful to check overlap regions and that the correct scale factor is being used.

```
plt.plot(red0[0].x, red0[0].y)
plt.plot(red1[0].x, red1[0].y)
#plt.plot(red2[0].x, red2[0].y)
plt.yscale('log')
plt.xscale('log')
```

The next steps print out the beam positions, actual angle of incidence, pixel size and sample to detector distance.

```
db0.direct_beam.m_beampos, db1.direct_beam.m_beampos, #db2.direct_beam.m_beampos
```

```
db0.reflected_beam.m_beampos, db1.reflected_beam.m_beampos, #db2.reflected_beam.m_beampos
```

```
reduction0['m_twotheta'][0, 0, 486:488], reduction0['omega'], reduction1['m_twotheta'][0, 0, 486:488], reduction1['omega
```

```
from refnx.util import q, angle, wavelength
angle(0.0102, 15.7), wavelength(0.01063, 0.65)
```

```
db0.direct_beam.cat.cat['qz_pixel_size'], db0.direct_beam.cat.cat['dy']
```

The data at this point is combined at the overlap region and the combined data can be plotted to ensure that the stitching is satisfactory.

```
combined_dataset = red0[0]
combined_dataset += red1[0]
#combined_dataset += red2[0]
```

```
plt.plot(combined_dataset.x, combined_dataset.y)
plt.yscale('log')
plt.xscale('log')
```

The final step in the reduction is to save the reduced data to file.

```
combined_dataset.save('c_SPZ0001039.dat')
```

However, one can reduce and combine all these files in a single step:

```
reduce_stitch([1039, 1041], [977, 978], prefix='SPZ', data_folder=data_folder, rebin_percent=3.0)
```

S3. Fitting parameters

Fitting was completed using the *refnx* fitting package (v0.1.18 onwards) originally developed by Nelson and Prescott (2019). The GUI application was used and instructions for use of the GUI can be found here: https://refnx.readthedocs.io/en/latest/, which also has links to download the latest version of *refnx*; here we use version 1.18. Supplementary Table S2 below describes the model files used for the fitting which can be loaded into the *refnx* GUI along with the corresponding reduced reflectivity data. The file contains the layers used to describe the system, and the parameters and their associated boundaries for each layer. Data were fitted using a differential evolution routine to minimise χ^2 values and uncertainties were determined using a MCMC algorithm. Models with multiple isotopic contrasts were co-refined simultaneously with the thickness and roughness constrained to be the same for each contrast.

Table S2 Description of the model files used for the fitting described in the manuscript.

File name	Description
coef_c_SPZ0000745.pkl	Bragg mirror at $\Delta\lambda/\lambda \sim 1\%$
coef_c_SPZ0000610.pkl	Bragg mirror at $\Delta\lambda/\lambda \sim 5\%$
coef_c_SPZ0000773.pkl	Bragg mirror at $\Delta\lambda/\lambda \sim 12\%$
coef_c_SPZ0000526.pkl	Silicon wafer
coef_c_SPZ0000581.pkl	Quartz wafer
coef_c_SPZ0000591.pkl	Sapphire wafer
coef_c_SPZ0000675.pkl	Spin coated d_8-polysyrene on silicon in air
coef_c_SPZ0000665.pkl	Silicon-D_2O
coef_c_SPZ0000667.pkl	Silicon-H_2O

coef_c_SPZ0003493.pkl	Spin coated h-polystyrene on silicon in D_2O
coef_c_SPZ0003495.pkl	Spin coated h-polystyrene on silicon in CM4
coef_c_SPZ0003498.pkl	Spin coated h-polystyrene on silicon in CMSi
coef_c_SPZ0003500.pkl	Spin coated h-polystyrene on silicon in H_2O
coef_c_SPZ0003473.pkl	DMPC bilayer on silicon at 37°C in D_2O
coef_c_SPZ0003474.pkl	DMPC bilayer on silicon at 37°C in CM4
coef_c_SPZ0003475.pkl	DMPC bilayer on silicon at 37°C in CMSi
coef_c_SPZ0003476.pkl	DMPC bilayer on silicon at 37°C in H_2O

S4. Supporting references

Nelson, A. R. J. & Prescott, S. W. (2019). *Journal of Applied Crystallography* **52**, 193-200.
